# Glymphatic System Dysfunction in Epilepsy: Clinical and Translational Perspectives

**DOI:** 10.1177/15357597261443067

**Published:** 2026-04-20

**Authors:** Dong Ah Lee, Ho-Joon Lee, Kang Min Park

**Affiliations:** 1 Department of Neurology, Haeundae Paik Hospital, 222187 Inje University College of Medicine, Busan, Republic of Korea; 2 Department of Radiology, Haeundae Paik Hospital, Inje University College of Medicine, Busan, Republic of Korea

**Keywords:** epilepsy, glymphatic system, neuroimaging, cognitive dysfunction

## Abstract

Epilepsy has traditionally been viewed as a disorder involving neuronal hyperexcitability and brain network dysfunction. However, growing evidence indicates that recurrent seizures are associated with widespread disturbances in brain homeostasis, including metabolic stress, neuroinflammation, vascular dysregulation, and sleep disruption. These processes extend beyond neurons and involve brain-wide clearance mechanisms that have received limited attention in epilepsy research. The glymphatic system is a specialized pathway that facilitates cerebrospinal fluid-interstitial fluid exchange and promotes the clearance of metabolic waste and neurotoxic solutes from the brain. Glymphatic transport depends on astrocytic aquaporin-4 channels and is strongly modulated by sleep-wake state, which is highly relevant to epilepsy given the close bidirectional relationship between seizures and sleep disturbances. Impaired glymphatic clearance has been linked to protein accumulation, neuroinflammation, and cognitive decline during aging and in neurodegenerative diseases, suggesting that similar mechanisms may contribute to epilepsy-related disease progression. In this review, we summarize current knowledge of glymphatic anatomy and physiology, focusing on advances in neuroimaging. We then synthesize emerging evidence demonstrating glymphatic dysfunction across multiple epilepsy syndromes. We discuss the clinical implications of impaired cerebral waste clearance for disease burden, treatment outcomes, and cognitive dysfunction and highlight potential therapeutic strategies aimed at modulating glymphatic function. Finally, we address the ongoing debates regarding glymphatic mechanisms, imaging biomarkers, and causal relationships in epilepsy. Collectively, the available data suggest that glymphatic system dysfunction represents a system-level abnormality in epilepsy, offering a complementary framework that integrates the metabolic, vascular, and sleep-related aspects of epileptic brain dysfunction.

## Glymphatic System Involvement in Epilepsy

Epilepsy has traditionally been conceptualized as a disorder involving neuronal excitability and brain network dysfunction. However, accumulating evidence indicates that seizures are not merely transient electrical events, but are accompanied by profound metabolic, vascular, and homeostatic disturbances within the brain. Recurrent seizures impose substantial metabolic stress, promote extracellular metabolite accumulation, disrupt sleep-wake architecture—processes that extend beyond neurons—and implicate non-neuronal clearance mechanisms.^1‐3^

The glymphatic system, a brain-wide pathway mediating cerebrospinal fluid (CSF)-interstitial fluid (ISF) exchange, is a key mechanism for metabolic waste clearance and solute distribution in the central nervous system. This system depends on astrocytic aquaporin (AQP)-4 water channels and is most active during sleep, a state often disrupted in patients with epilepsy. Because sleep deprivation is a well-established seizure trigger and many epilepsies involve fragmented or abnormal sleep, impaired glymphatic function may be an underrecognized contributor to epileptogenesis and seizure persistence.^4‐6^

Importantly, glymphatic clearance failure has been linked to neurotoxic protein accumulation, neuroinflammation, cognitive decline during aging, and neurodegenerative disorders. These pathological features substantially overlap with the chronic consequences of epilepsy, including progressive cognitive dysfunction and pharmacoresistance. Despite these conceptual parallels, the role of the glymphatic system in epilepsy has only recently been explored and remains poorly understood.^7,8^

In this review, we summarize current knowledge of the glymphatic system and its imaging in humans, examine emerging evidence linking glymphatic dysfunction to epilepsy, and discuss potential clinical implications and therapeutic strategies. We also address the ongoing debates regarding the existence, mechanisms, and measurement of glymphatic flow, highlighting the critical gaps in epilepsy research.

### Overview of the Glymphatic System

The glymphatic system is a macroscopic waste-clearance pathway that facilitates the exchange of CSF and ISF throughout the brain parenchyma. Unlike peripheral tissues, the central nervous system lacks conventional lymphatic vessels despite its high metabolic demand. The glymphatic system compensates by using perivascular spaces formed by astrocytic endfeet to enable convective fluid movement and solute clearance.^6,9‐11^

CSF is primarily produced by the choroid plexus and circulates through the ventricular system into the subarachnoid space. CSF enters the brain along the periarterial (Virchow-Robin) spaces surrounding the penetrating arteries. These spaces are externally bound by astrocytic endfeet that express highly polarized AQP4 channels, which are preferentially localized to the perivascular endfeet membranes rather than the astrocytic soma or processes. This polarization is maintained by anchoring complexes involving dystrophin-associated proteins, which concentrate AQP4 at the astrocytic endfeet adjacent to the vasculature, thereby facilitating directional water transport between perivascular spaces and the interstitium.^12^ As arteries branch into arterioles and capillaries, the perivascular spaces become narrow and continuous with the basal lamina, allowing the CSF to penetrate the brain parenchyma.^6,9‐11^ Within the parenchyma, CSF mixes with ISF and drives convective solute transport toward the perivenous spaces. ISF is subsequently cleared along the veins and ultimately drains into the cervical lymphatic pathways. This directional flow, from periarterial influx to perivenous efflux, distinguishes the glymphatic system from simple diffusion-based exchange.^6,9‐11^ Current paradigms increasingly regard the glymphatic pathway as functionally coupled with meningeal lymphatic vessels, forming an integrated central nervous system lymphatic network. This expanded framework positions brain waste clearance within a continuous drainage hierarchy that ultimately connects the central nervous system to the peripheral lymphatic system.^13^

Multiple physiological mechanisms drive glymphatic transport. Arterial pulsatility plays a central role in propelling CSF along periarterial spaces, while respiration and CSF pressure gradients further contribute to fluid movement. AQP4 channels facilitate high water permeability across astrocytic endfeet, effectively reducing transmembrane resistance at the interface between perivascular spaces and the interstitial compartment. In the absence of AQP4, astrocytic membranes represent a significant barrier to water movement; however, dense perivascular localization of AQP4 enables rapid bidirectional water flux, thereby lowering hydraulic resistance and supporting pressure-driven convective flow. This structural arrangement allows efficient coupling between perivascular CSF movement and interstitial fluid exchange, rather than relying solely on slow diffusion. Loss of AQP4 polarization increases resistance at this interface and impairs CSF-ISF exchange.^6,9‐11^

A defining feature of the glymphatic system is its strong dependence on the behavioral state: activity is markedly enhanced during sleep and suppressed during wakefulness. Experimental studies have demonstrated up to 90% reduction in CSF influx during wakefulness compared to sleep, a difference attributed to the norepinephrine-mediated regulation of interstitial space volume and CSF dynamics. During sleep, expansion of the interstitial space facilitates convective flow and promotes the efficient clearance of metabolic waste.^6,9‐11^

In addition to waste removal, the glymphatic system supports brain-wide distribution of biologically important compounds, including glucose, lipids, neuromodulators, and potential drugs. Astrocytes play a dual role by synthesizing lipid carriers, such as apolipoprotein E, and maintaining the perivascular pathways that enable solute transport.^6,9‐11^

Glymphatic function declines with age and is impaired in conditions such as traumatic brain injury, stroke, and neurodegenerative diseases. These impairments are associated with the loss of perivascular AQP4 polarization, reduced arterial pulsatility, and altered CSF production. Collectively, these observations suggest that the glymphatic system is a critical interface between vascular, glial, and metabolic processes that is highly relevant to epilepsy pathophysiology.^6,9‐11^

### Brain Imaging of the Glymphatic System

In rodent models, glymphatic transport is typically visualized using intracisternal or intrathecal injection of fluorescent or radiolabeled tracers. High-resolution two-photon microscopy enables real-time imaging of tracer movement along periarterial pathways and into the interstitial space at the microscopic level, providing real-time visualization of CSF-ISF exchange dynamics. In addition, fluorescent tracer studies combined with ex vivo imaging of brain sections allow high-resolution mapping of tracer distribution throughout the parenchyma, enabling quantitative assessment of glymphatic transport at the whole-brain level. Radiotracer-based approaches have also been used to evaluate tracer clearance kinetics, providing complementary information on glymphatic function for assessing glymphatic transport in vivo.^4,5^ In contrast, direct visualization of glymphatic transport in the human brain remains challenging, as it requires invasive tracer-based approaches unsuitable for routine or large-scale studies and operates at slow velocities within perivascular and interstitial spaces. Consequently, most studies rely on magnetic resonance imaging (MRI)-based surrogate markers rather than direct measurement of CSF-ISF flow. These approaches can be broadly categorized as tracer-based imaging and non-invasive contrast-free techniques.^14,15^

Intrathecal administration of gadolinium-based contrast agents is currently the closest approximation to a gold standard for assessing glymphatic transport in humans. Serial MRI following lumbar intrathecal injection demonstrates brain-wide periarterial influx, delayed parenchymal enhancement, and subsequent redistribution toward the perivenous pathways, meningeal lymphatic vessels, and perineural drainage routes along the cranial and spinal nerves. Prolonged tracer retention has been observed in conditions such as idiopathic normal pressure hydrocephalus and sleep deprivation, supporting impaired glymphatic clearance in these states.^15,16^ However, intrathecal contrast administration requires lumbar puncture and remains off-label. Although recent studies suggest that the procedure is generally safe and feasible, short-term adverse events and limited long-term safety data constrain its widespread clinical applicability. Intravenous contrast-enhanced MRI using heavily T2-weighted fluid-attenuated inversion recovery sequences provides a less invasive alternative. Such approaches have been used to visualize meningeal lymphatic structures, parasagittal dural enhancement patterns, and ocular glymphatic pathways. Dynamic imaging strategies, including longitudinal T1 mapping, have enabled the observation of delayed signal changes in brain structures following intravenous gadolinium administration. However, because intravenous techniques are influenced by blood-brain barrier permeability, vascular dynamics, and contrast redistribution, they are best regarded as indirect rather than direct measures of glymphatic transport.^15,17‐19^

Given these limitations, non-invasive MRI surrogate markers have attracted increasing attention. Diffusion tensor imaging analysis along the perivascular space (DTI-ALPS) is the most widely used technique for estimating water diffusivity along perivascular pathways, exploiting the geometric relationship between the medullary veins and surrounding white matter tracts. Reduced DTI-ALPS indices have been reported in aging, neurodegenerative diseases, sleep disorders, and cerebrovascular conditions, and correlate with cognitive performance and amyloid burden.^15,20,21^ Nevertheless, DTI-ALPS measures water diffusion rather than solute clearance and is restricted to specific white matter regions. Its sensitivity to head positioning, region-of-interest selection, and the underlying white matter microstructure raises important questions regarding its specificity as a global glymphatic marker. A range of MRI-based techniques investigate the distinct structural and physiological components of brain fluid dynamics. Free-water imaging estimates the extracellular fluid content, intravoxel incoherent motion modeling reflects microvascular perfusion-related diffusion, and perivascular space volumetry captures the structural correlates of impaired interstitial drainage. Therefore, these methods assess specific elements of the broader neurofluid system rather than directly quantifying global glymphatic clearance, and most lack temporal resolution for tracking dynamic solute transport.^15,21^ Advanced MRI approaches targeting CSF motion, including phase-contrast imaging, time-spatial labeling inversion pulse techniques, arterial spin labeling-based water exchange metrics, and ultrafast functional MRI, interrogate distinct physiological components of the neurofluid system, such as pulsatile CSF flow, respiratory coupling, and global brain-CSF interactions. In humans, large-amplitude CSF oscillations synchronized with slow-wave sleep underscore the state-dependent regulation of neurofluid dynamics, although the relationship between oscillatory CSF movements and net solute clearance remains debatable. Emerging techniques further aim to visualize meningeal lymphatic structures directly and to quantify trans-barrier water exchange, potentially reflecting AQP-mediated transport under conditions of intact blood-brain barrier integrity. These methods highlight the fact that contemporary glymphatic imaging assesses the discrete components of a distributed clearance network rather than a single unified process.^22,23^

Collectively, the current imaging approaches capture complementary but incomplete aspects of glymphatic function. No single modality provides a comprehensive assessment of brain-wide clearance, and fluid movement does not necessarily correlate with solute transport. The lack of methodological standardization remains a major barrier to clinical translation, underscoring the need for multimodal and harmonized imaging strategies.

### Glymphatic System Dysfunction in Epilepsy

Experimental evidence further supports a causal relationship between epilepsy and glymphatic dysfunction. In rodent models of status epilepticus, glymphatic transport is significantly suppressed during the acute phase and remains impaired in the chronic epileptic state. Pharmacological enhancement of glymphatic flow attenuates seizure severity and suppresses epileptogenesis, whereas genetic deletion of AQP4, a key regulator of perivascular fluid transport, exacerbates seizure burden and prolongs epileptic activity.^5,24,25^ These findings indicate that disruption of astrocytic AQP4 polarization and perivascular fluid dynamics plays a central role in epilepsy progression. Experimental and translational studies further suggest that impaired glymphatic clearance may promote the accumulation of pro-inflammatory cytokines and neurotoxic metabolites, contributing to a pro-epileptogenic microenvironment characterized by neuroinflammation, blood-brain barrier dysfunction, and neuronal hyperexcitability.^5,24‐26^

Accumulating evidence from human neuroimaging studies indicates that dysfunction of the glymphatic system is a common feature of various epilepsy syndromes. Recent investigations using diffusion-based and structural MRI surrogates have consistently demonstrated impaired CSF-ISF exchange in patients with epilepsy, suggesting that altered cerebral waste clearance is a fundamental pathophysiological component rather than a syndrome-specific epiphenomenon.^27,28^

Most human studies assessing the glymphatic function in epilepsy have relied on DTI-ALPS indices. Across a wide range of epilepsy syndromes, including juvenile myoclonic epilepsy, newly diagnosed focal epilepsy, temporal lobe epilepsy with hippocampal sclerosis, occipital lobe epilepsy, and status epilepticus, DTI-ALPS indices are consistently lower in patients than in healthy controls. These findings indicate the presence of widespread alterations in perivascular water diffusivity beyond the epileptogenic zones.^27,29‐31^

Studies using perivascular space enlargement as a marker have reported converging evidence of glymphatic dysfunction in patients with epilepsy. Although both reduced DTI-ALPS indices and enlarged perivascular spaces are considered markers of glymphatic impairment, they reflect different aspects of the system. DTI-ALPS indices primarily reflect alterations in water diffusivity along perivascular pathways, whereas perivascular space enlargement represents structural and drainage-related changes within the perivascular compartment. Therefore, lower DTI-ALPS indices do not necessarily predict reduced or increased perivascular space burden, and these measures should be interpreted as complementary rather than directly correlated indicators of glymphatic dysfunction. Enlargement and increased burden of the perivascular spaces, particularly within the basal ganglia and deep white matter, have been reported in both focal and generalized epilepsies.^32,33^ Notably, glymphatic impairment has also been demonstrated even in patients with normal conventional MRI findings. Studies on MRI-negative epilepsy, including pediatric populations, have revealed reduced perivascular diffusivity accompanied by increased interstitial free-water content, suggesting defective clearance rather than primary white matter degeneration.^34^

Although focal epilepsy is often conceptualized as a disorder confined to a localized epileptogenic network, glymphatic abnormalities frequently extend beyond the seizure-onset zones. Several imaging studies have demonstrated bilateral or widespread reductions in glymphatic surrogate markers, even in patients with unilateral epileptogenic foci, suggesting that recurrent seizures may induce global disturbances in neurovascular and glial homeostasis rather than isolated focal effects.^27,29,30^ Repetitive ictal and interictal activity is associated with blood-brain barrier disruption, which permits the influx of plasma-derived proteins and inflammatory mediators into the perivascular and interstitial spaces. Such leakage may lead to the local accumulation of serum proteins at sites of barrier disruption, increasing the burden on glymphatic clearance pathways. Although direct evidence for this process in focal epilepsy remains limited, experimental and clinical studies have shown that blood-brain barrier breakdown is associated with protein extravasation, neuroinflammation, and impaired clearance mechanisms. This altered microenvironment may increase resistance to interstitial fluid movement and interfere with CSF-ISF exchange.^35,36^ In parallel, seizure-related neuroinflammatory signaling and astrocytic remodeling can disrupt perivascular architecture, including loss of AQP4 polarization at astrocytic endfeet, which further impairs convective fluid transport.^1^ Additionally, seizure-induced alterations in vascular tone and arterial pulsatility—key driving forces of periarterial CSF influx—may further compromise glymphatic transport. Together, these mechanisms provide a biological framework linking focal epileptic activity to distributed impairment of cerebral waste clearance pathways, reframing epilepsy as a disorder that disrupts system-level neurofluid regulation in addition to neuronal excitability.^29,37^

Taken together, current human imaging data support the concept that epilepsy is associated with impaired cerebral waste clearance. Rather than reflecting an isolated regional pathology, glymphatic dysfunction appears to represent a shared network-level abnormality across epilepsies, highlighting that epilepsy involves disruption of neurofluid regulation in addition to neuronal excitability.

### Clinical Implications of Glymphatic System Dysfunction in Epilepsy

#### Age, Disease Duration, and Seizure Burden

Across multiple epilepsy cohorts, glymphatic system dysfunction was consistently associated with the cumulative disease burden. Human imaging studies have demonstrated inverse relationships between glymphatic surrogate markers, particularly the DTI-ALPS indices, and patient age, epilepsy duration, and seizure burden.^27,30^

In both adult and pediatric epilepsy, a longer disease duration is associated with a progressive reduction in glymphatic efficiency, even after adjusting for demographic variables. These findings suggest that glymphatic impairment reflects long-term exposure to recurrent epileptic activity rather than a static predisposing trait.^29,34^ A higher seizure frequency has similarly been associated with reduced glymphatic indices, supporting the notion that repeated neuronal hyperexcitability disrupts perivascular fluid dynamics. Mechanistically, recurrent seizures may promote blood-brain barrier dysfunction, astrocytic swelling, and loss of AQP4 polarization, all of which compromise CSF-ISF exchange.^37,38^ Importantly, these relationships are likely bidirectional. While seizure activity may impair glymphatic transport, a reduced clearance capacity may facilitate the accumulation of neurotoxic metabolites and inflammatory mediators, thereby lowering seizure thresholds over time. This feed-forward relationship positions glymphatic dysfunction as both a marker and a contributor to epilepsy progression.^28^

#### Medical and Surgical Outcome

Beyond reflecting the disease burden, glymphatic system dysfunction has emerged as a clinically relevant imaging correlate of treatment outcomes in epilepsy. Several studies have reported that patients with poor medical or surgical outcomes exhibit more pronounced impairment in glymphatic surrogate markers.^28^

In medically treated epilepsy, reduced glymphatic function has been observed in patients with persistent seizures compared to those achieving seizure control. Impaired perivascular transport may influence the tissue-level pharmacokinetics of anti-seizure medications or limit the clearance of seizure-promoting metabolites, although causality remains unproven.^38,39^

Surgical cohorts provide particularly compelling evidence. In temporal lobe epilepsy, glymphatic indices are frequently reduced ipsilateral to the epileptogenic focus. Following successful anterior temporal lobectomy, partial recovery of glymphatic function has been demonstrated, suggesting that ongoing epileptic activity actively suppresses perivascular fluid dynamics.^40,41^ Recent studies have further indicated that glymphatic-related markers, such as choroid plexus enlargement and altered coupling between global blood oxygen level-dependent signals and CSF inflow, independently predict postsurgical outcomes. These findings highlight the importance of brain-wide neurofluid regulation beyond focal structural pathology.^42^

### Cognitive Function

Cognitive impairment is a common disabling comorbidity of epilepsy. Emerging evidence suggests that glymphatic system dysfunction contributes to cognitive decline by impairing metabolic waste clearance, promoting neuroinflammation, and disrupting large-scale brain networks.^28^

Across focal epilepsies, particularly temporal lobe epilepsy, reduced glymphatic function assessed using DTI-ALPS indices is correlated with poor memory performance, even after controlling for hippocampal volume. In patients with hippocampal sclerosis, bilateral glymphatic impairment is associated with more severe memory deficits, whereas non-lesional temporal lobe epilepsy often shows lateralized dysfunction with relatively preserved cognition, unless the impairment becomes widespread.^29,43^ Associations extend beyond the temporal lobe. In frontal lobe epilepsy, reduced glymphatic function correlates with executive dysfunction and working memory impairment, suggesting that impaired perivascular fluid dynamics disrupt frontal network efficiency.^30^ Structural components of the glymphatic system further reinforce this relationship. Enlargement of the choroid plexus correlates with lower global cognitive scores and widespread gray matter atrophy, with mediation analyses implicating hippocampal and thalamic degeneration.^42^

In pediatric epilepsy, an increased perivascular space burden has been associated with reduced intelligence quotient and disrupted structural network efficiency, emphasizing the role of glymphatic pathways in brain development and cognitive resilience.^34,44^ In older patients with late-onset epilepsy, glymphatic dysfunction correlates with global cognitive decline and amyloid biomarkers, highlighting the convergence between epilepsy-related cognitive impairment and neurodegenerative processes.^45^

Collectively, these findings support glymphatic dysfunction as a network-level contributor to cognitive impairment in patients with epilepsy.

### Therapeutic Perspectives

The recognition of the glymphatic system as a modifiable brain-wide clearance pathway provides new therapeutic perspectives for epilepsy. Although direct glymphatic-targeted therapies remain largely experimental, converging evidence suggests that enhanced glymphatic function may influence epileptogenesis, seizure-related injury, and long-term outcomes.^46^

Astrocytic AQP4 is a central target. Disruption of AQP4 polarization impairs glymphatic transport and contributes to neuronal hyperexcitability and potassium dysregulation in experimental models. Restoration of astrocytic endfoot integrity, rather than indiscriminate upregulation of AQP4, has therefore emerged as a rational therapeutic strategy.^47^ The glymphatic-meningeal lymphatic continuum offers another promising avenue. The enhancement of meningeal lymphatic drainage improves waste clearance and cognitive performance in preclinical models, suggesting its potential relevance in epilepsy-associated neuroinflammation.^48^

Non-pharmacological strategies may offer immediate translational relevance. Sleep optimization is particularly attractive given the strong state dependence of glymphatic transport. Sleep deprivation impairs clearance and promotes the accumulation of epileptogenic metabolites, supporting structured sleep interventions as indirect glymphatic modulators.^49^ Pharmacological approaches targeting inflammation, vascular integrity, and metabolic support may exert secondary benefits on glymphatic function, although epilepsy-specific evidence remains limited.^50^

Overall, glymphatic modulation should be viewed as complementary to conventional anti-seizure strategies, addressing the downstream consequences of recurrent seizures rather than replacing seizure control therapies.

### Controversies Surrounding Glymphatic Function in Epilepsy

Despite the growing interest, substantial conceptual and methodological debates remain. A central concern involves the interpretation of diffusion-based metrics, such as the DTI-ALPS indices, which reflect directional water diffusivity rather than direct CSF-ISF exchange. Alterations in DTI-ALPS indices may therefore reflect nonspecific white matter or vascular changes rather than primary glymphatic dysfunction.^15,20,51^ This limitation is underscored by findings of altered DTI-ALPS indices in functional neurological disorders, including psychogenic non-epileptic seizures, raising questions regarding disease specificity.^52^ Similarly, structural markers such as Virchow-Robin space enlargement should not be directly equated with glymphatic failure, as perivascular space dilation is influenced by multiple factors—including blood pressure fluctuations, inflammation, developmental stage, CSF pressure dynamics, and microvascular compliance—rendering it a non-specific indicator of broader neurovascular fluid alterations. Moreover, postictal reductions in perivascular space burden may reflect resolution of acute edema or normalization of CSF dynamics rather than true restoration of glymphatic flow, which would require corroboration using complementary approaches such as tracer-based imaging, phase-contrast CSF flow measurements, or AQP4-related biomarkers.^53^ A substantial body of experimental evidence supports the notion that glymphatic transport is enhanced during sleep, particularly under conditions of reduced noradrenergic tone and increased interstitial space volume. However, a recent tracer-based study has suggested that glymphatic influx may be reduced during sleep under certain experimental conditions, although these findings remain preliminary and have not yet been consistently replicated.^54^ The apparent discrepancy may reflect differences in experimental design, anesthetic conditions, or the specific components of glymphatic transport being assessed. Taken together, these findings highlight an ongoing debate, although the prevailing evidence still favors enhanced glymphatic activity during sleep. Finally, most human epilepsy studies have been cross-sectional, precluding causal inference. It remains unclear whether glymphatic dysfunction contributes to epileptogenesis or reflects accumulated brain injury.

In summary, glymphatic system research provides a compelling framework linking epilepsy to impaired brain homeostasis; however, careful interpretation and methodological rigor are essential. Multimodal imaging, physiological measurements, and longitudinal design are critical for defining the true role of cerebral waste clearance pathways in epilepsy.
